# Supplementary material for: Heavy Vacuum Gas Oil Upregulates the Rhamnosyltransferases and Quorum Sensing Cascades of Rhamnolipids Biosynthesis in Pseudomonas sp. AK6U
Source: Molecules. 2021 Jul 6;26(14):4122. doi: 10.3390/molecules26144122 (PMC8307569; doi:10.3390/molecules26144122)
Supplement: Supplementary file 1 [file molecules-26-04122-s001.zip › molecules-1222838-supplementary.pdf]

Supplementary Materials

Heavy Vacuum Gas Oil Upregulates the Rhamnosyltransferases and Quorum Sensing Cascades of Rhamnolipids Biosynthesis in *Pseudomonas* sp. AK6U

Sarah A. Alkhalaf [1], Ahmed R. Ramadan [2], Christian Obuekwe [3], Ashraf M. El Nayal [1], Nasser Abotalib [1] and Wael Ismail [1,*]

1 Environmental Biotechnology Program, Life Sciences Department, College of Graduate Studies, Arabian Gulf University, Manama 26671, Bahrain; soz-82@hotmail.com (S.A.A.); ashrafmt@agu.edu.bh (A.M.E.N.); nasseraa@agu.edu.bh (N.A.)
2 Health Biotechnology Program, Life Sciences Department, College of Graduate Studies, Arabian Gulf University, Manama 26671, Bahrain; ahmedrr@agu.edu.bh
3 Department of Biological Sciences, Faculty of Science, Kuwait University, Kuwait 12037, Kuwait; okeyobuekwe@hotmail.com
* Correspondence: waelame@agu.edu.bh; Tel: +973-36146948

Table S1. Preparation of chemically defined medium from stock solutions.

Stock Solution (Concentration)	Volume Added (mL) Per litter of Medium
Phosphate buffer (1M)	50 mL
NH_4Cl (1M)	10 mL
$MgSO_4.7H_2O$ (1M)	1 mL
$CaCl_2.2H_2O$ (0.3 M)	1 mL
Trace elements	1 mL
$FeSO_4.7H_2O$	1 mL
Vitamin solution (VL7)	1 mL
Distilled H_2O	Up to 1 L

Trace elements solution (g/500 mL, 1000-fold)

$ZnSO_4.7H_2O$	0.075
$MnSO_4.H_2O$	0.0425
$CuSO_4.5H_2O$	0.0185
$CoCl_2.6H_2O$	0.1
$NiCl_2.6H_2O$	0.01
$Na_2MoO_4.2H_2O$	0.02
H_3BO_3	0.01

Vitamins solution (mg/250 mL, 1000-fold)

Cyanocobalamine	25
Pyridoxine-HCl	75
Thiamin-HCl	50
Nicotinic acid	50
p-Aminobenzoate	40
Biotin	40
Ca-Pantothenate	25

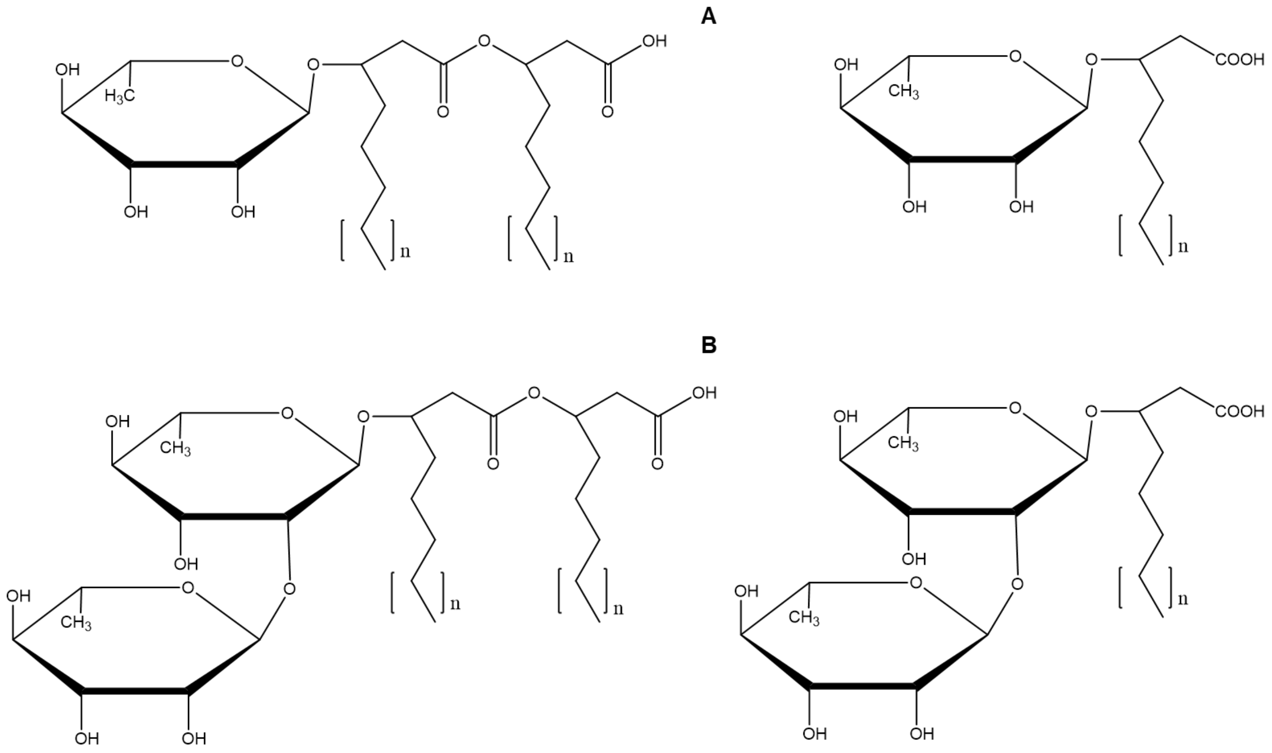

Figure S1. Chemical structures of mono- (**A**) and di-(**B**) rhamnolipids.

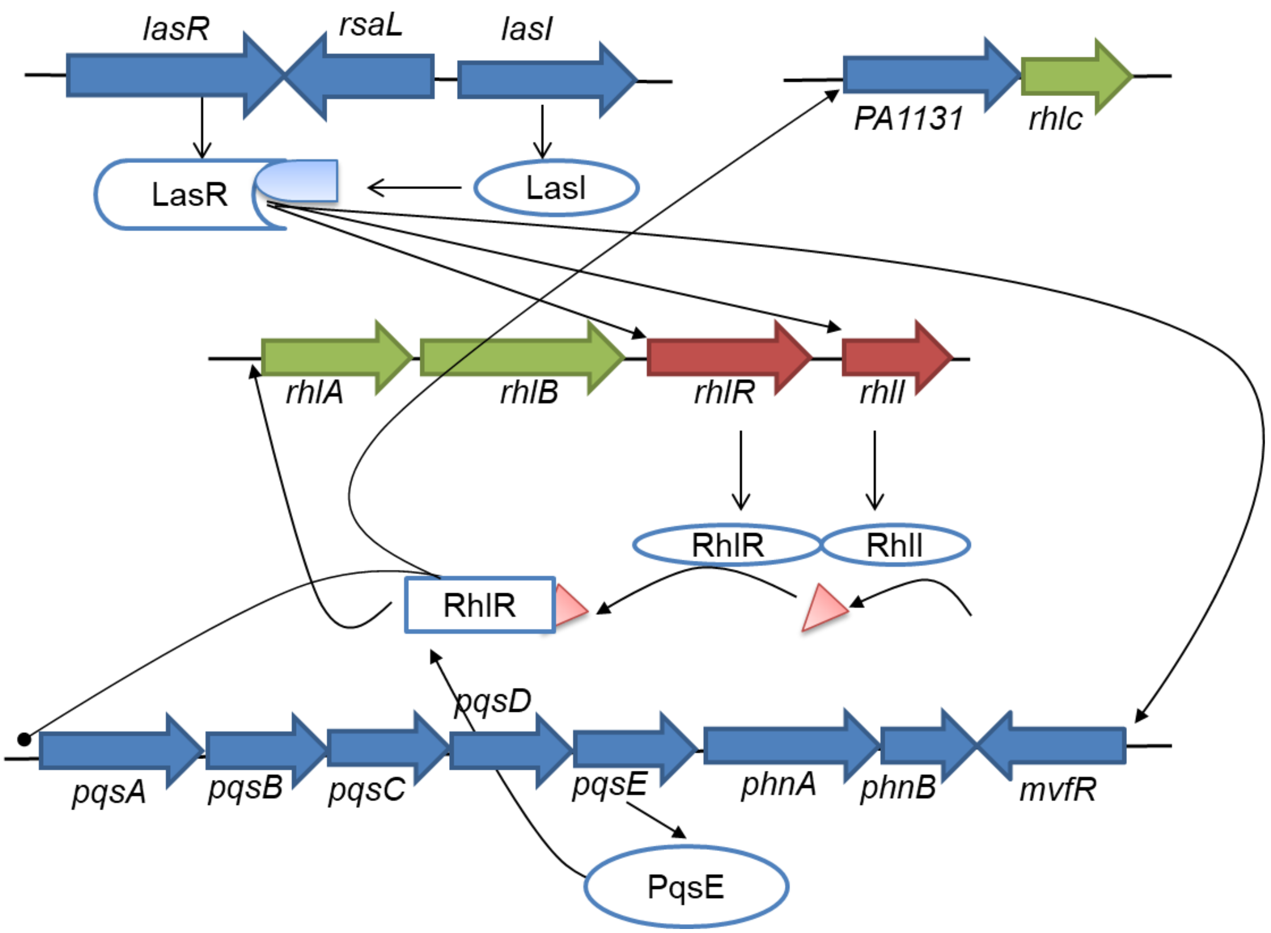

Figure S2. Genes of rhamnolipids biosynthesis and the involved quorum-sensing networks in *P. aeruginosa*. Two quorum-sensing systems, LasR/I and RhlR/I, are involved in the regulation of rhamnolipids biosynthesis. The LasR/I system depends on the acyl homoserine lactones (AHL) N-3-oxododecanoyl-HSL (3-oxo-C_{12}-HSL), while the RhlR/I system operates with N-butanoyl-HSL (C_4-HSL). The HSL autoinducers or ligands are produced by the autoinducer synthases LasI and RhlI and then bind to their cognate transcriptional regulators, LasR and RhlR, respectively, which regulate the rhamnolipids biosynthesis genes. LasR/I and RhlR/I activate the expression of their own autoinducer synthase genes, *lasI* and *rhlI*, respectively, as a positive feedback. RhlR/C_4-HSL complex works by positively regulating the expression of the thamnolipids biosynthesis genes *rhlABC*. LasR/3-oxo-C_{12}-HSL activates the other quorum sensing system, in which the transcriptional regulator MvfR (PqsR) binds to its co-inducers 4-hydroxy-2-heptylquinoline (HHQ) and 2-heptyl-3-hydroxy-4-quinolone (*Pseudomonas* Quinolone Signal; PQS). The PQS signaling molecule is produced by the PqsABCD proteins, whereas HHQ production requires PqsABCD in addition to the protein encoded by *pqsH*, which is located elsewhere in the chromosome. LasR/3-oxo-C_{12}-HSL activates the expression of *mvfR* [8].

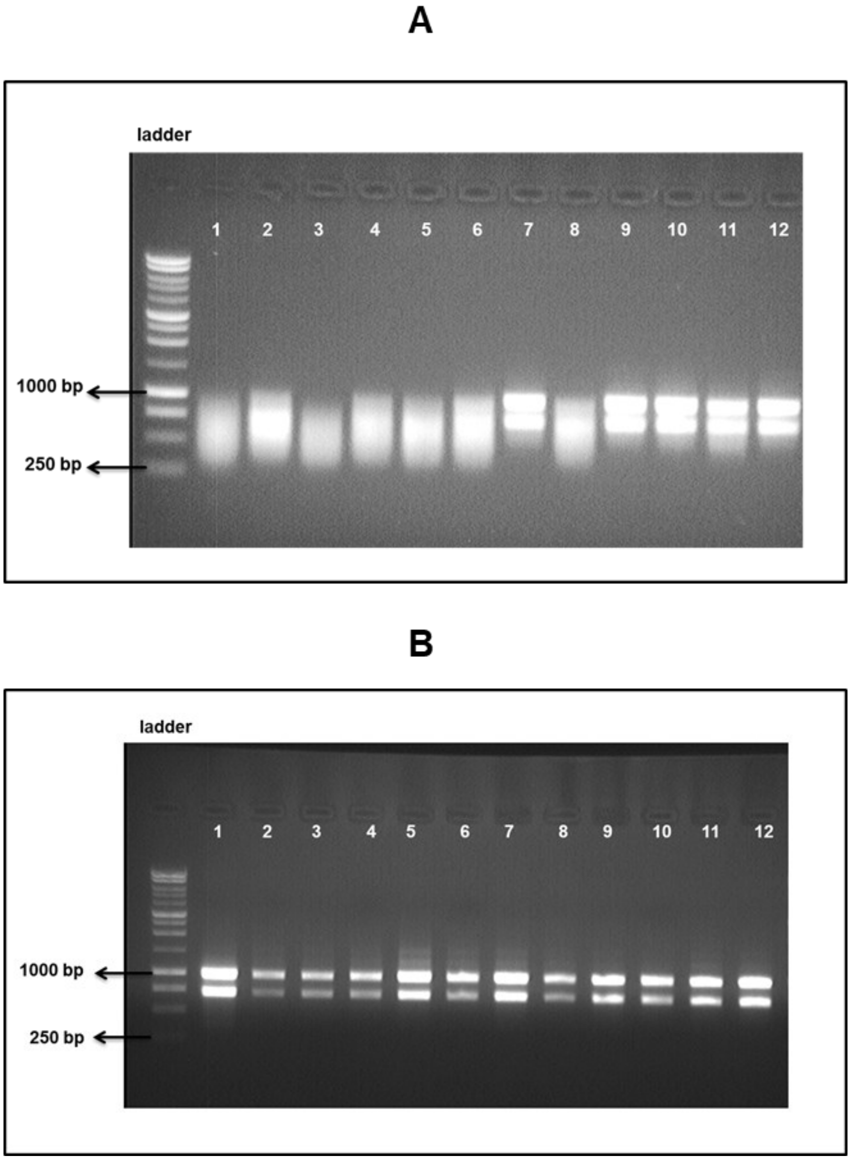

Figure S3. Samples of total RNA isolated from the glucose (**A**) and HVGO (**B**) cultures of the AK6U strain at the early (lanes 1-6) and late (lanes 7-12) log phases of growth.

A

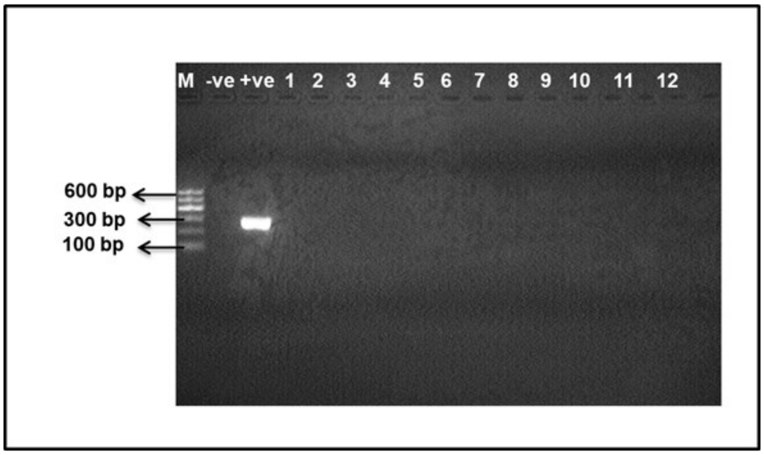

B

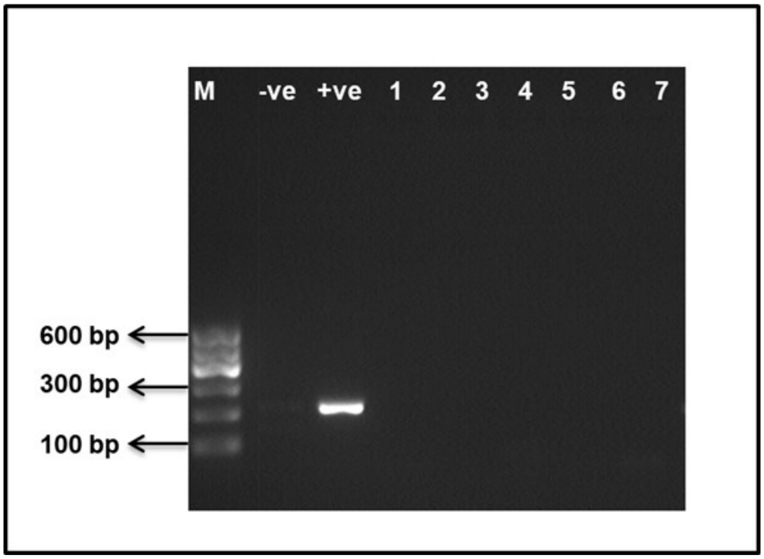

Figure S4. PCR was performed to check for genomic DNA contamination in the RNA isolated from the glucose (**A**) and HVGO (**B**) cultures. The PCR product is a fragment of the *rhlA* gene (263 bp) amplified with the primers RhlA-F and RhlA-R. M, DNA marker; -ve, no-template control; +ve, a positive control assay using genomic DNA from the AK6U strain as a template.

A

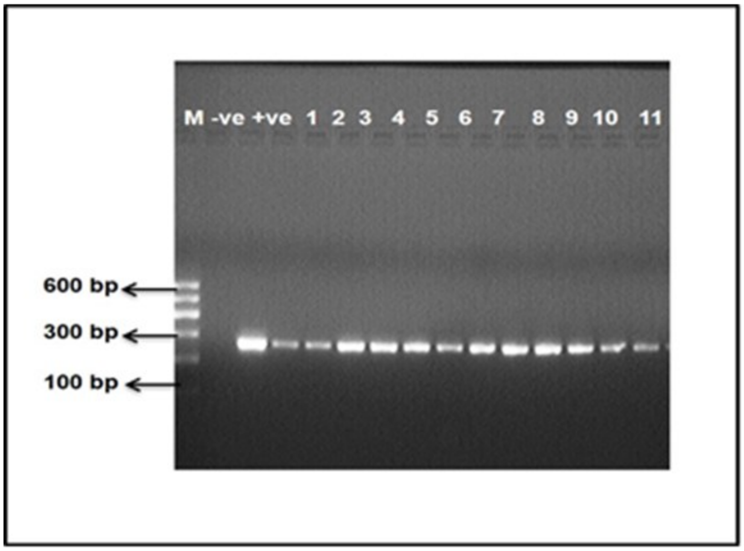

B

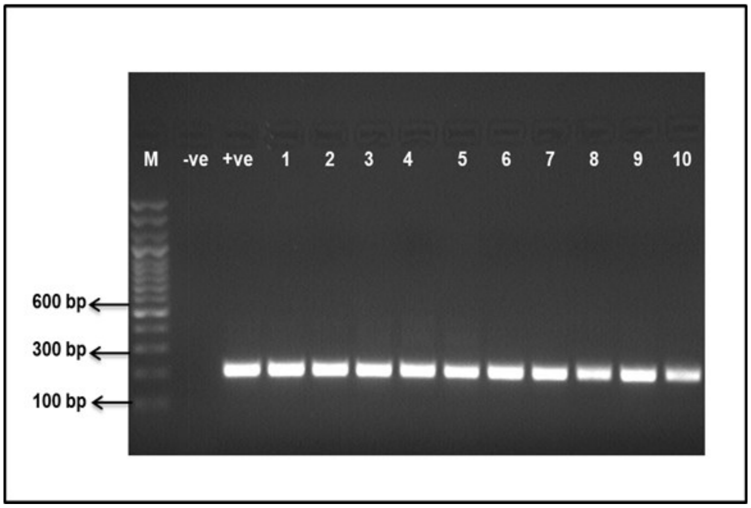

Figure S5. A fragment (263 bp) of the *rhlA* gene amplified from cDNA of the glucose (**A**) and HVGO (**B**) cultures with the primers RhlA-F and RhlA-R. M, DNA marker; -ve, no-template control; +ve, a positive control assay using genomic DNA from the AK6U strain as a template.

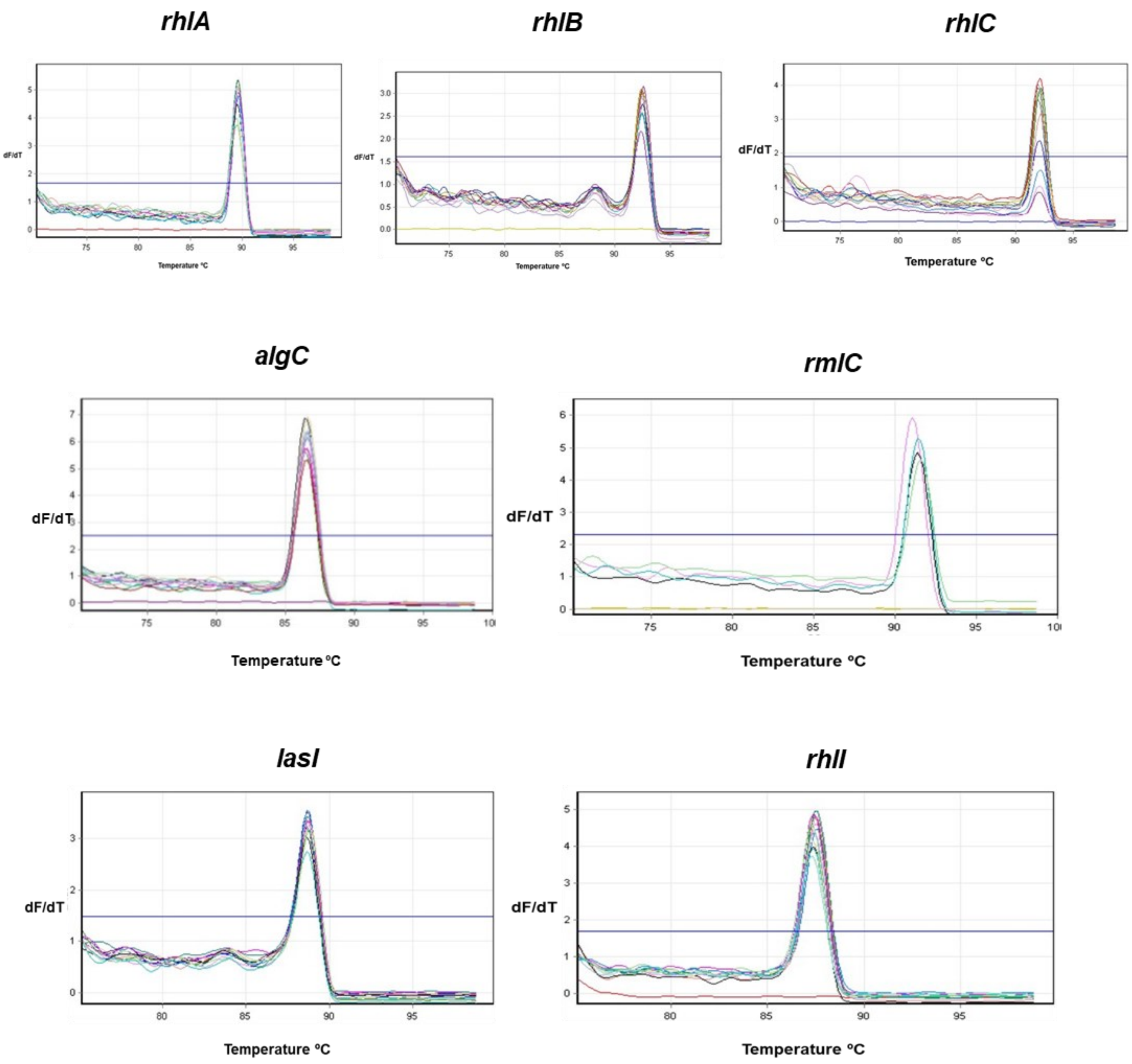

Figure S6. Melt curve analysis.
